# The mechanism driving a solid–solid phase transition in a biomacromolecular crystal

**DOI:** 10.1107/S2052252521004826

**Published:** 2021-06-17

**Authors:** Saminathan Ramakrishnan, Jason R. Stagno, William F. Heinz, Xiaobing Zuo, Ping Yu, Yun-Xing Wang

**Affiliations:** a Structural Biophysics Laboratory, Centre for Cancer Research, National Cancer Institute, Frederick, MD 21702, USA; b Optical Microscopy and Analysis Laboratory, Cancer Research Technology Program, Frederick National Laboratory for Cancer Research, Frederick, MD 21702, USA; c X-ray Science Division, Argonne National Laboratory, Lemont, IL 60439, USA

**Keywords:** solid–solid phase transition mechanisms, time-resolved crystallography, RNA structural biology, large conformational changes

## Abstract

Solid–solid phase transitions (SSPTs) occur between distinguishable crystalline forms. Because of their importance in application and theory in materials science and condensed-matter physics, SSPTs have been studied most extensively in metallic alloys, inorganic salts and small organic molecular crystals, but much less so in biomacromolecular crystals. In general, the mechanisms of SSPTs at the atomic and molecular levels are not well understood. Here, the ordered molecular rearrangements in biomacromolecular crystals of the adenine riboswitch aptamer are described using real-time serial crystallography and solution atomic force microscopy. Large, ligand-induced conformational changes drive the initial phase transition from the apo unit cell (AUC) to the trans unit cell 1 (TUC1). During this transition, coaxial stacking of P1 duplexes becomes the dominant packing interface, whereas P2–P2 interactions are almost completely disrupted, resulting in ‘floating’ layers of molecules. The coupling points in TUC1 and their local conformational flexibility allow the molecules to reorganize to achieve the more densely packed and energetically favorable bound unit cell (BUC). This study thus reveals the interplay between the conformational changes and the crystal phases – the underlying mechanism that drives the phase transition. Using polarized video microscopy to monitor SSPTs in small crystals at high ligand concentration, the time window during which the major conformational changes take place was identified, and the *in crystallo* kinetics have been simulated. Together, these results provide the spatiotemporal information necessary for informing time-resolved crystallography experiments. Moreover, this study illustrates a practical approach to characterization of SSPTs in transparent crystals.

## Abstract 2

The mechanism of a solid–solid phase transition in an RNA crystal is presented.

## 1. Introduction   

In both organic and inorganic crystals, rearrangements of atoms or small molecules occur in diffusionless solid–solid phase transitions (SSPTs), which are generally triggered by temperature, humidity, pressure or mechanical stress (Commins *et al.*, 2016 ▸; Anwar & Zahn, 2017 ▸). SSPTs are often classified as first or second order according to the proposal of Ehrenfest (Porter *et al.*, 2008 ▸; Pogatscher *et al.*, 2016 ▸; Peng *et al.*, 2015 ▸; Mnyukh, 2009 ▸). From general thermodynamic studies of oscillators to current investigations on neural oscillations in the brain (Brochini *et al.*, 2016 ▸), SSPTs have been a topic of great interest. Well known examples are phase changes in shape memory alloys caused by temperature variation (Otsuka & Wayman, 1998 ▸), or in small organic molecular crystals by heat or light exposure (Etter & Siedle, 1983 ▸; Skoko *et al.*, 2010 ▸; Smets *et al.*, 2020 ▸). Notably, in shape memory alloys, the challenge between increased entropy and intrinsic order in a crystal drives the molecular rearrangement to overcome the heat applied (Everhardt *et al.*, 2019 ▸). SSPTs have also been observed in living systems with functional roles, for example, in strain-induced tail-sheath contraction in T-even bacteriophages (Olson & Hartman, 1982 ▸; Harris & Scriven, 1970 ▸) and in flagella-assisted bacterial locomotion (Baker *et al.*, 2006 ▸; Calladine, 1978 ▸). However, mechanisms of SSPTs in organic and inorganic crystals at the molecular and atomic levels are relatively poorly understood due to technical challenges, but frequently involve dislocation generation and motion. A very recent study of organic molecular crystals illustrates the cooperative motion in phase transitions (Smets *et al.*, 2020 ▸).

The SSPT is a dynamic process that requires the existence of a solid phase (*e.g.* a crystal) throughout. Unlike solid–liquid phase transitions, for instance in lipid membranes, SSPTs typically progress through movements of atoms restricted to within a few ångstroms, and are governed collectively by intermolecular interactions. Unfortunately, as a result, use of crystallographic methods alone to study phase transitions of single macromolecular crystals has not been possible, as they rely on trapping crystals in various transitional states rather than real-time observations, and/or are restricted to systems involving only small conformational changes. Moreover, if there is any dissolution of crystals during phase transition, the reversible assembly of a macromolecular crystal to solid intermediate or final phase is hypothetical in a liquid environ­ment. Solution atomic force microscopy (AFM) and polarization video microscopy (PVM) methods exclude this possibility through continuous monitoring of a single crystal throughout the SSPT.

Riboswitches are versatile, functional RNA elements that undergo conformational transitions upon ligand binding to regulate gene expression (Nahvi *et al.*, 2002 ▸). Previous studies using mix-and-inject time-resolved serial crystallography (TRX) revealed that the addition of a ligand to crystals of adenine riboswitch aptamer RNA (riboA) result in large conformational changes that drive the crystals through an SSPT, from monoclinic (AUC), to triclinic (TUC1), to orthorhombic (BUC) (Stagno *et al.*, 2017 ▸; Ramakrishnan, Stagno, Conrad *et al.*, 2021 ▸). The structure of AUC contains two unique conformers (apo1/apo2) of riboA in the asymmetric unit. The binding of the ligand Ade leads to accumulation of [apo1/IB·Ade], which eventually triggers the AUC–TUC1 lattice transition through ligand-driven rotation and translation of the P1 helix by 15–22° and 8 Å, respectively. Upon completing the transition to TUC1, both molecules exhibit the bound-like ([B·Ade]-like) conformation. Eventually, the [B·Ade]-like molecules in TUC1 reorient to achieve the optimal packing through distinct lattice-coupling points, thus forming [B·Ade] in the ortho­rhombic BUC setting. The lattice reorganization from TUC1 to BUC likely involves the TUC2 intermediate lattice, but further studies are needed to elucidate its involvement in the transition pathway. Importantly, since the biologically relevant conformational changes occur in the AUC–TUC1 transition, understanding the mechanism of this stage of the SSPT and the time-window during which it takes place is paramount for guiding subsequent TRX experiments for mapping out a molecular movie. Here, we present a detailed analysis of the SSPT in riboA crystals and the underlying mechanism that drives the transition. Using AFM and PVM, we directly visualized, in real time, the ordered lattice changes and molecular rearrangements throughout the SSPT. The atomic and molecular details during the transition at each step are further corroborated with TRX data, which provide atomic structures of riboA and its crystal contacts at each stage of the SSPT. Our *in situ* studies reveal, at the molecular and atomic levels, the interplay between the molecular conformational changes and their resultant lattices, which is the underlying mechanism that drives the synchronous phase transition in riboA crystals.

## 2. Methods   

### 2.1. Structural analysis of crystal packing interfaces   

The crystal structures of riboA in AUC [PDB entry 5e54 (Stagno *et al.*, 2017 ▸)], TUC1 [PDB entry 6vwt (Ramakrishnan, Stagno, Conrad *et al.*, 2021 ▸)] and BUC [PDB entries 5swe (Stagno *et al.*, 2017 ▸) and 4tzx (Zhang & Ferre-D’Amare, 2014 ▸)] were analyzed using the *PyMOL* Molecular Graphics System (Schrödinger, LLC). As there were no major discernable differences between the TRX and single-crystal BUC structures (PDB entries 5swe and 4tzx, respectively), the higher resolution and higher quality structure (PDB entry 4tzx) was used throughout for depiction of the BUC model. Multiple unit cells were generated by symmetry in the *ab* or *ac* plane using the ‘supercell’ *PyMOL* extension. All unique contact interfaces for a single molecule were identified and interacting residues in each interface were defined as those involved in hydrogen bonding (≤3.5 Å) or van der Waals (≤4.0 Å) interactions. For AUC, in which the two molecules of the asymmetric unit are structurally unique, both molecules were included in the identification of packing interfaces. The buried surface area (BSA) for each interface was calculated as (*A* + *B* − *A* *B*)/2, where *A* and *B* are the individual solvent accessible surface areas (SASA) of each interacting molecule *A* and *B* in isolation, respectively, and *A* *B* is the SASA of the two molecules together, using a solvent radius of 1.4 Å.

### 2.2. Mica surface-assisted riboA crystal growth   

The crystallization buffer consisted of 40 m*M* sodium cacodylate, pH 6.5, 80 m*M* KCl, 100 m*M* MgCl_2_, 12 m*M* spermine tetra­hydro­chloride and 32–65%(*v*/*v*) 2-methyl-2,4-pentane­diol (MPD). To prepare the stabilization buffer, equal volumes of crystallization buffer (65% MPD) and RNA buffer (10 m*M* HEPES pH 7.5, 100 m*M* KCl, 0.5 m*M* EDTA) were mixed. The buffers were stored at room temperature and homogenized before use. A freshly cleaved mica wafer was first treated with 10 m*M* spermidine for 15 min. After treatment, the mica was washed with ultrapure water and air-dried. The mica was then adhered to an EasyXtal crystallization support (Qiagen, Germantown, MD, USA) using a drop of paraffin oil. Next, 0.5 µl of 1:1000 dilution of crystal seed solution was placed in the center of the mica wafer and incubated for 1 min. The sample was covered tightly with a glass box to avoid evaporation. 2.5 µl of gel-purified riboA was mixed with an equal volume of crystallization buffer (32% MPD), and immediately and gently dispensed onto the mica containing the seed solution. The sample, on the support, was then inverted and tightly screwed into a well in the EasyXtal crystallization plate, suspended over 700 µl of crystallization buffer. The vapor-diffusion crystallization setup was incubated overnight at 22°C.

### 2.3. AFM imaging   

AFM topographs of the crystals were collected using a Cipher VRS AFM (Asylum Research, Oxford Instruments, Santa Barbara, California, USA) fitted out with a temperature control and a cantilever holder with integrated perfusion ports. The mica substrate with riboA crystals was first fixed on a stainless-steel puck (Ted Pella, Redding, CA, USA) by transparent ep­oxy resin. To avoid evaporation, the sample was tightly covered using a Petri dish after 20–30 µl of stabilization buffer was added on the surface. The sample was then placed on a magnetic sample stage, and the probe holder was connected via tubing to the inlet and outlet buffer reservoirs. A total of 100 µl of stabilization buffer was carefully infused through the perfusion tubes to cover the mica surface. The temperature of the AFM stage was set to 15°C, and the sample temperature was equilibrated in the AFM chamber for at least 30 min prior to measurements. The intermittent contact mode was used to image the crystal surface using a BL-AC40TS-C2 Biolever mini probe (Asylum Research, Oxford Instruments, Santa Barbara, CA, USA). The resonance frequency (∼12 kHz) of the probe in stabilization buffer was obtained using the thermal peak method. The crystal surfaces were measured at a line scan rate of 12 Hz, with a scan size of 256 × 256 pixels (100 × 100 nm). After the AUC topography was acquired, the ligand-free buffer was carefully removed and exchanged with 150 µ*M* (*ac* plane) or 50 µ*M* (*ab* plane) adenine ligand in stabilization buffer (∼500 µl). The imaging continued until the BUC phase was recorded. Due to the ligand-induced physical changes in the crystal, the contact between the probe and the crystal surface was frequently lost. However, the setpoint was adjusted to recover the contact, and imaging continued using the parameters already recorded.

### 2.4. AFM image processing in SPIP and unit-cell dimensions calculation   

Due to the viscosity of the stabilization buffer and soft sample surface, the raw AFM data show very low signal-to-noise. *Scanning Probe Image Processor* software (*SPIP*) (Image Metrology, Lyngby, Denmark) was used to reduce the noise and obtain ultra-sharp topographs. First, the AFM data pixel resolution was set to 2024 × 2024. Then, the fast fourier transform (FFT) function was used to remove any random noise by manually picking prominent FFT peaks and generating filtered images via the inverse FFT. The corresponding autocorrelation function of filtered images was used to determine the unit-cell dimensions by manual inspection. Figs. S1 and S2 of the supporting information show the original AFM image, corresponding filtered image and autocorrelation function of AUC, TUC1 and BUC in both the *ac* and the *ab* planes. The AFM images are 40 × 40 nm (*ac* plane) or 30 × 30 nm (*ab* plane) in size. Table S4 shows the unit-cell dimensions of each phase, derived from the autocorrelation function.

### 2.5. Superimposition of crystal structures onto the AFM images   

**Figure 1:**
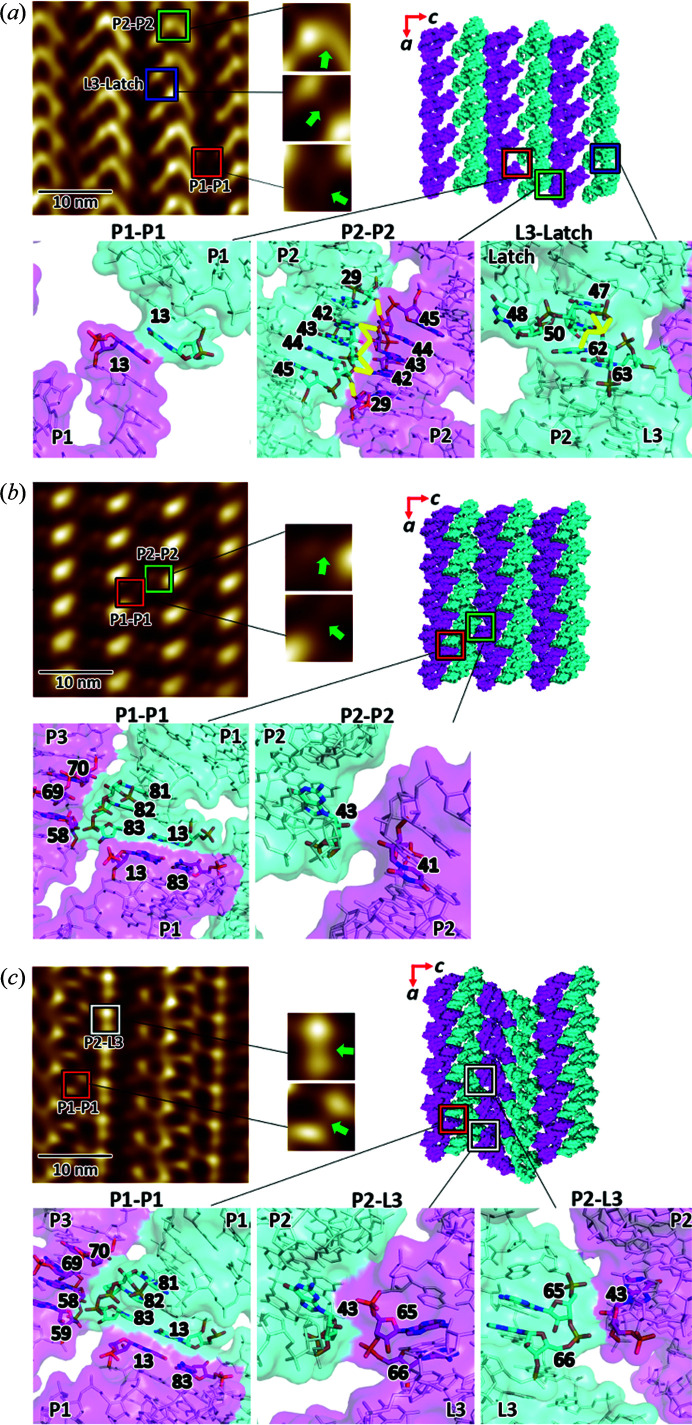
Crystal packing in the *ac* plane of (*a*) AUC (PDB entry 5e54), (*b*) TUC1 (PDB entry 6vwt) and (*c*) BUC (PDB entry 4tzx), as observed by continuous AFM of the same crystal, and the associated structures determined by X-ray crystallography. The molecular interfaces (P2–P2, P1–P1, P2–L3 and L3–latch) in each phase are highlighted with colored boxes, and respective zoomed-in views are shown. The atomic inter­actions at each interface are shown as sticks with a transparent molecular surface. Hydrogen-bond interactions are drawn as yellow lines. The unit-cell dimensions derived from X-ray diffraction or AFM are listed in Tables S3 and S4. AFM images are 30 × 30 nm in size and 0.5 nm in height (dark-to-light color scale). Ligand concentrations for AFM and TRX experiments were 0.15 m*M* and 10 m*M*, respectively.

Using the unit-cell dimensions of AUC, TUC1 and BUC obtained from X-ray diffraction and AFM images, the orientations of the crystal structures relative to the AFM images were determined. The crystal structures, containing several molecules in the *ac* or *ab* plane, were superimposed onto the AFM images, guided by topographical features. First, the crystal structure image transparency was reduced to 55% and placed on the AFM image. An approximate superimposition was carried out manually by extending the crystal structure to fit the unit cell in the AFM image. Then, the placement of molecules was taken into consideration to correlate the crystal structure with the AFM image. For instance, both the crystal structure and the AFM image of the AUC *ac* plane exhibit two molecules closely stacked [Fig. 1 ▸(*a*)] in a column, which corresponds to the P2–P2 interaction. The crystal structure was tilted and adjusted accordingly to match the orientation in the AFM image. The same method was applied to TUC1 and BUC images of the *ac* plane [Figs. 1 ▸(*b*) and 1(*c*)]. In particular, the BUC *ac* plane has every pair of adjacent columns rotated by 180°. Such features aid in maximizing the overall fit of the overlayed images.

### 2.6. PVM experiment to record ligand-induced SSPTs in riboA crystals   

An optically transparent glass-bottom dish coated with poly d-lysine (MatTek Corporation, P35GC-1.5–14-C) was used to grow riboA crystals directly on the surface. To initiate crystal growth, 0.5 µl of 1:1000 dilution riboA crystal seed solution was placed in the middle of the glass-bottom dish. The seed solution was allowed to adsorb to the coated surface for 1 min. To avoid evaporation, the sample was tightly covered with a glass beaker. Then, 5 µl of an equal mixture of gel-purified riboA and 34% MPD crystallization buffer was gently placed on the seed solution. The crystallization setup was then flipped upside down and fixed on the dish cover containing 700 µl of 34% MPD crystallization buffer and tightly sealed with Teflon tape. The crystallization setup was kept at 22°C for 12 h. Before the PVM experiment, the glass bottom dish with crystals was first rinsed with 34% stabilization buffer to remove the excess RNA. The dish was then filled with 1.5 ml 34% stabilization buffer, fixed on a PVM stage and covered with a glass cover slip. The rectangular *ac*-face crystals 5–10 µm in size were selected, centered and focused. The angle of polarization was optimized to observe the intensity changes due to crystal birefringence. A volume of 1.5 ml 20 m*M* adenine in 34% stabilization buffer was added to the dish (final adenine concentration of 10 m*M*) while the crystal was continuously recorded. To avoid evaporation, the sample was carefully covered with a glass cover slip. The video of intensity changes in the crystal throughout the ligand-induced SSPT was recorded at 2456 pixels × 1842 pixels and 200 ms exposure time for 132 s, with a frame rate of ∼275 ms per frame. Time-lapsed image data were visualized, processed and analyzed with *FIJI* (Schindelin *et al.*, 2012 ▸). In brief, AVI files were imported into *FIJI* as a z-stack of 16-bit TIFFs and converted to greyscale. The crystal dimensions were measured manually with the *FIJI* line tool. A region of interest (ROI) (Fig. 4, green square), corresponding to 80 × 80 pixels (∼2 × 2 µm, 24.75 nm per pixel), was selected for analysis, cropped and saved as a greyscale AVI file. A custom *MatLab* (v. 2019b, Mathworks) script, as described by Ramakrishnan, Stagno, Magidson *et al.* (2021 ▸), was used to measure the intensity of birefringence as a function of time and its first derivative. For the analysis, the 80 × 80 pixel ROI was spatially averaged by 10 × 10 pixels resulting in an 8 × 8 pixel ROI with 246 nm per pixel resolution, the approximate diffraction-limited optical resolution of PVM. The kinetics simulation methods for the T1 transition are described in the supporting information.

### 2.7. AFM imaging of forward and reverse SSPTs in riboA crystals   

The riboA crystals were grown on mica surface using the same methodology described above. Before the ligand infusion, the AUC topography was recorded several times to identify the optimum measurement criteria for AFM. Approximately 100 µl of 50 µ*M* adenine ligand in stabilization buffer was infused gently inside the AFM sample stage while the crystal surface was continuously imaged. The forward SSPT was carried out at 15°C. When the final BUC topography was recorded, the imaging was stopped, and 1 ml of ligand-free stabilization buffer was infused through the perfusion tubes and completely exchanged with the ligand-containing stabilization buffer. This buffer exchange was carried out several times and the crystal sample was finally soaked in the ligand-free buffer overnight at 15°C. After 12 h of soaking, the buffer in the sample stage was replaced with 1 ml of fresh stabilization buffer, and the temperature of the experimental setup was increased to 35°C. Every 30 min, 1 ml of fresh buffer was infused inside the chamber and this process was continued for (at least) a further 5 h. The AUCrev topography was recorded after 17–20 h. The AFM topographs of forward and reverse SSPT phases were then processed in *SPIP* using the protocol described above.

## 3. Results   

### 3.1. Crystal packing viewed from the *ac* plane   

The ordered multiphasic transition in the riboA crystals is driven by synchronous ligand-induced conformational changes and their resultant intermolecular lattice coupling points. Maintaining crystalline order throughout the SSPT is therefore dependent on the distinct crystal packing inter­actions at each stage, and the coordinated changes between them. The lattice changes are most visible in the *ac* plane (Figs. 1 ▸ and 2 ▸). The molecular arrangement in AUC observed by AFM is consistent with the crystal structure [Fig. 1 ▸(*a*)] which, when superimposed onto the AUC AFM topography [Fig. S3(*a*)], highlights the distinct crystal packing interfaces. The molecules in the *ac* plane of AUC are coupled predominantly through a symmetrical network of hydrogen bonds and van der Waals (VDW) contacts involving residues 29 and 42–45, located in the P2 stems of adjacent molecules [Fig. 1 ▸(*a*), bottom].

In the AFM image, the P2–P2 interface is prominent and is observed between the columns of stacked riboA molecules along the *a* axis [Fig. 1 ▸(*a*)]. The smaller interface, which includes residues 47 and 50 of one molecule, and 62 of another (L3-latch), is also visible. However, there is a very weak packing interaction between the unstable P1 helices via base-stacking of terminal residues of opposing 5′-strands [Fig. 1 ▸(*a*), bottom]. This dynamic interface is mostly invisible to AFM, most likely owing to its flexibility and partial disorder, but is somewhat apparent in the far-right column [Fig. 1 ▸(*a*)]. Large available spaces around the P1 helices indicate that, in the AUC phase, these regions are highly accommodating to structural changes.

**Figure 2:**
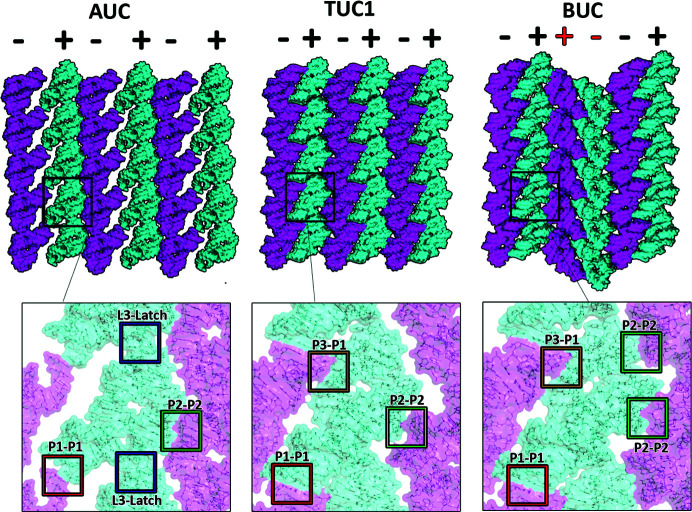
Comparison of the orientations of riboA molecules in the *ac* plane of each crystal phase, AUC (left), TUC1 (middle) and BUC (right), and their respective crystal packing interfaces. The lattice plane can be described as columns of riboA molecules, each of which has an associated direction (+ or −) based on the way the molecules are facing. Red + and – highlight the change in orientation of the columns in the BUC phase, relative to AUC/TUC1.

Continuous AFM imaging of the crystal surface after the addition of ligand reveals the SSPT to form TUC1 [Fig. 1 ▸(*b*)]. The ligand-stabilized helices solidify the P1–P1 coaxial stacking interface in TUC1, which is further stabilized by VDW interactions between the 3′ terminus and residues 58, 69 and 70 of two adjacent molecules [Fig. 1 ▸(*b*)]. This effectively reduces the distance between adjacent molecules along *c* by ∼1.5 nm [9.3 nm (AUC) to 7.8 nm (TUC1); Figs. 1 ▸(*a*) and 1(*b*)]. In contrast to AUC, TUC1 crystal packing is dominated by the P1–P1 interaction, whereas the P2–P2 interface becomes almost negligible. Both interfaces are observed in the AFM image [Fig. 1 ▸(*b*)]. The extensive P2–P2 interface in AUC is minimized in TUC1 to a VDW contact between residues 41 and 43, thereby resulting in ‘floating’ layers of molecules along this planar interface. Furthermore, as residue 48 is displaced by the ligand and flips out into the solvent, the L3–latch interface is altered, and contributes more to stacking in the *ab* plane [compare Figs. 1(*a*) ▸ and 3(*b*) ▸]. In general, the latch and kissing-loop regions (in particular residues 48, 35–36 and 62–64) are more dynamic in TUC1. Such freedom of motion amongst every other layer of molecules in TUC1 drives the transition to BUC, as the molecular layers reorganize to achieve a lower lattice energy [Fig. 1 ▸(*c*)]. The net result is that every other ‘pair’ of layers along the *a* axis is inverted (rotated 180° around *c*) (Fig. 2 ▸).

The stable P1–P1 interface is virtually unchanged from TUC1 to BUC, the only addition being residue 59 moving into a VDW distance of <4 Å [Fig. 1 ▸(*c*)]. The minimal P2–P2 interface [buried surface area (BSA): 84.5 Å^2^] in TUC1 now becomes a slightly stronger P2–L3 VDW interface (BSA: 117.5 Å^2^) involving residues 43 and 65–66 (Table S1). Moreover, because of the additional crystal symmetry, the P2–L3 interface is duplicated, providing even more coupling points. Given the conformational and lattice restraints, this is the most energetically favorable packing that can be achieved (Table S1). Interestingly, the AFM image of BUC in the *ac* plane [Fig. 1 ▸(*c*)] shows that the P1 helices of all molecules in a column are embedded, whereas other helical domains appear as prominent protrusions. This is consistent with the topographical staggering along the *b* axis, where P1 helices are below the lattice plane, relative to the remaining structure. The alternating arrangements of the molecular columns in BUC are consistent with the crystallographic observation that every other pair of columns is inverted [Figs. 1 ▸(*c*) and 2 ▸], indicating synchronous rotation and translation of molecules. The fact that the lattice coupling in BUC is only moderately enhanced relative to TUC1, yet requires seemingly significant molecular reorganization, may account for the extensive amount of time (∼3 h at 150 µ*M* ligand) it takes for the SSPT to reach BUC. In a similar manner, the crystal lattice can be reverted back to AUC by the removal of the ligand (Fig. S4, Table S2). The reversibility of the transition, again without entering the liquid phase, has important implications for the mechanism of this SSPT, which is controlled by conformational changes induced by the binding or unbinding of the ligand.

### 3.2. Crystal packing viewed from the *ab* plane   

**Figure 3:**
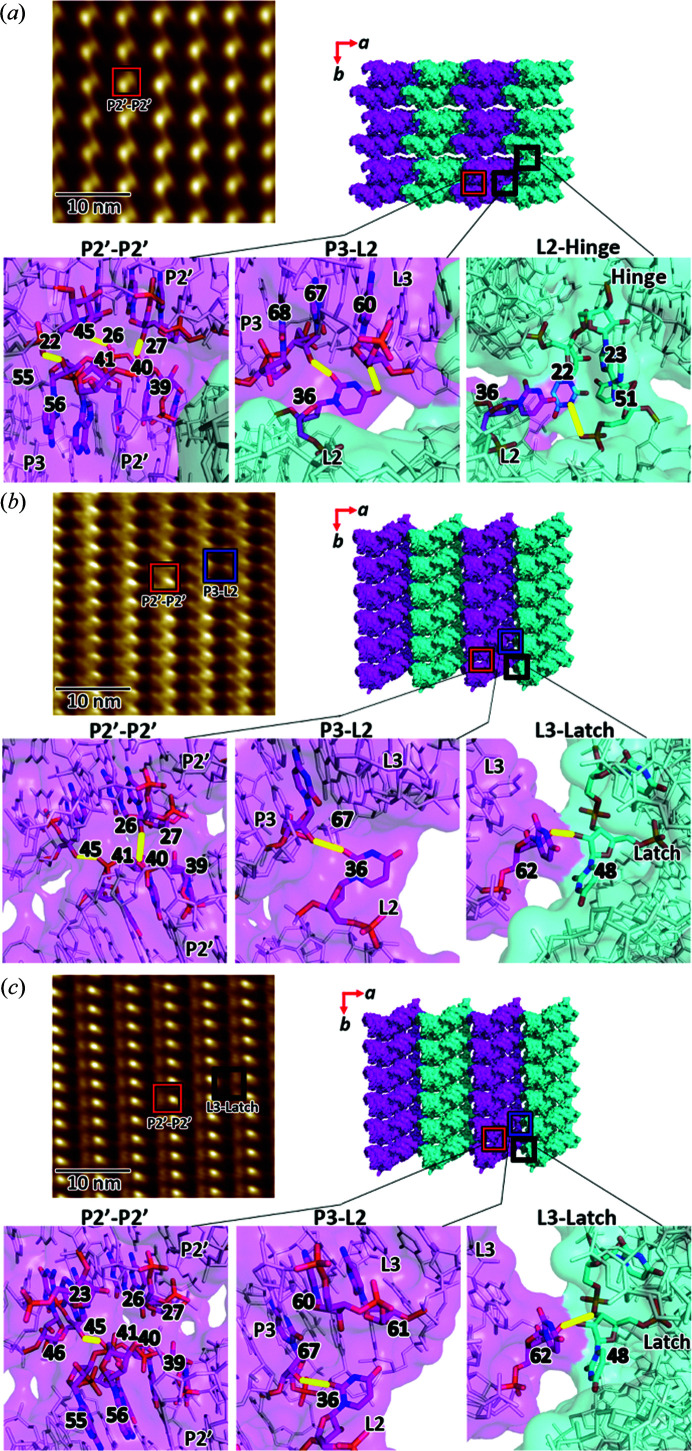
Crystal packing in the *ab* plane of (*a*) AUC, (*b*) TUC1 and (*c*) BUC, as observed by continuous AFM of the same crystal, and the associated structures determined by X-ray crystallography. The molecular interfaces (P2′–P2′, P3–L2, L3–latch) observed in AFM images are highlighted with colored boxes. The atomic interactions at each interface are shown as sticks with transparent molecular surfaces. Hydrogen-bond interactions are drawn as yellow lines. The unit-cell dimensions derived from X-ray diffraction or AFM are listed in Tables S3 and S4. AFM images are 30 × 30 nm in size and 0.5 nm in height (dark-to-light color scale). Ligand concentrations for AFM and TRX experiments were 0.05 and 10 m*M*, respectively.

We also examined the lattice transition in the *ab* plane, which is relatively conserved throughout the SSPT (Fig. 3 ▸). The persistent stacking of *ac* layers serves primarily to maintain lattice integrity, rather than promoting molecular re­organization, thereby preventing the crystal from entering the liquid phase. On average, the BSA for a molecule is ∼50% greater in the *ab* plane than in the *ac* plane (Table S1). The primary *ab* interface consists of hydrogen bonds and VDW contacts between the P2 helices (P2′–P2′), but in a slightly different region than the P2–P2 interface in the *ac* plane [Figs. 1 ▸(*a*) and 3 ▸(*a*)]. In AUC, P2′–P2′ involves residues 22, 26–27 and 45 of one molecule, and 39–41 and 55–56 of an adjacent molecule [Fig. 3 ▸(*a*)]. Strangely, the AFM image of the AUC *ab* plane reveals large voids between ‘doublets’ of protrusions. Upon inspection of the *ab* layer in the crystal structure, one side of the *ab* plane is relatively flat, whereas the other side exhibits significant height differentials between adjacent molecules. From this analysis, it can be inferred that the protrusion doublet corresponds to the P1 helices, all of which are oriented in the same direction. In TUC1, the P2′–P2′ interface is mostly maintained, except that the VDW contacts with P3 residues 55–56 are lost [Fig. 3 ▸(*b*)]. In BUC, the P2′–P2′ interface is more similar to that in AUC, but with fewer hydrogen bonds [Fig. 3 ▸(*c*)].

The second conserved *ab* interface involves P3 and L2. In particular, the hydrogen bond between residues 36 and 67 is maintained throughout the SSPT. In AUC, however, residue 36 forms an additional hydrogen bond with residue 60. AUC and BUC both exhibit more VDW interactions in the P3–L2 interface than in TUC1, similar to the trend observed in the *ac* plane, a feature of TUC1 that is perhaps essential for liberating the molecules enough to reorganize. A third coupling point in the AUC lattice exists between L2 of the other (unique) molecule in the asymmetric unit of AUC (L2′) and residues 22–23 (hinge) and 51 (latch) of two adjacent molecules, respectively. However, the interface is extremely weak as these residues reside in flexible regions with a high degree of disorder, and completely reconfigure upon ligand binding. In particular, the positions of distal atoms in residues 22, 23, 48 and 51 change by 8–19 Å upon conversion to TUC1. The weakness of this interface, therefore, is highly significant. Were these residues to be artificially stabilized by extensive crystal packing, the ligand-binding energy might be insufficient to drive the SSPT. Lastly, TUC1 and BUC both exhibit a unique *ab* interface between the latch (residue 48) and L3 (residue 62) [Figs. 3 ▸(*b*) and 3(*c*)], which is reminiscent of that observed in the *ac* plane of AUC [Fig. 1 ▸(*a*)]. The base-pairing with the uracil base of residue 48, however, is not possible in AUC, as this residue occupies the ligand-binding site in the apo conformation.

The AFM image of the AUC *ab* plane [Fig. 3 ▸(*a*)] shows that the molecules are in a square (equidistant) arrangement, as the *a* and *b* unit-cell dimensions of AUC are almost identical. In contrast, *ab* images of TUC1 and BUC reveal the halving of *b* (from ∼4.8 to ∼2.5 nm). The persistence of the P2′–P2′ interface in all three lattices is evident in the AFM images as columns of stacked molecules along *b*, as is the rotation of molecules in TUC1 and BUC, relative to AUC. However, correlating crystallographic arrangements in the *ab* plane with AFM topography is more challenging than in *ac*. This is due to the changing of cell angles (α and β), which manifests as a tilt of the plane of molecules relative to the viewing angle. In the *ac* plane, only the change in α has this effect, as changes in β are simply movements in the plane itself. In the *ab* plane, however, both α and β influence the relative viewing angle. This is evident when comparing the *ab* planes of TUC1 and BUC. For visual purposes and analysis, the single layer of molecules in each crystal structure [Figs. 3 ▸(*b*), 3(*c*) (right)] was made flat, even though the viewing axis may not be perfectly normal to that plane. As a result, the packing arrangements in the *ab* plane of the TUC1 and BUC crystal structures appear identical. However, α changes from ∼99 (TUC1) to 90° (BUC), thereby changing the relative viewing angle by ∼9°. By comparing the AFM images of these two surfaces [Figs. 3 ▸(*b*) and 3(*c*) (left)], it is evident that, even though the layer contains roughly the same molecular arrangement, the change in the relative viewing angle results in a slightly different topography.

### 3.3. Temporal characterization of the SSPT   

**Figure 4:**
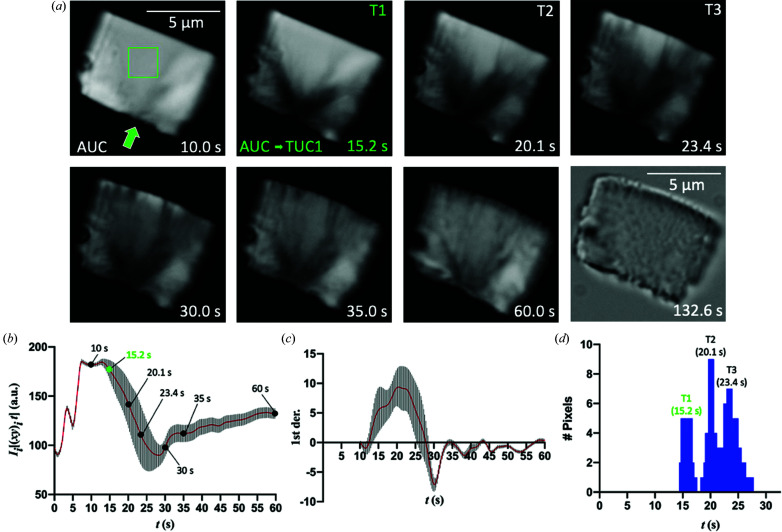
(*a*) PVM images of a riboA crystal (8 × 6 µm) in the presence of 10 m*M* adenine ligand. A crystal defect, which serves as the point of origin for the phase transition, is indicated by the green arrow. (*b*) Averaged intensity of crystal birefringence for each spatially averaged (10 × 10 pixels) superpixel in the selected ROI (80 × 80 pixels, green square) plotted versus time. (*c*) The first derivative of the intensity plot in (*b*). (*d*) Histogram of the time values of the first-derivative peaks for all superpixels (64), revealing T1, T2 and T3. As the addition of ligand causes disturbance to birefringence intensity and view of the crystal under the microscope, measurements prior to 10 s were omitted from the analysis.

The first stage of the SSPT (AUC to TUC1) is driven by the ligand-induced major conformational changes in [apo1/apo2] (AUC) to form [B·Ade]-like × 2 (TUC1). Surprisingly, many of these changes include residues directly involved in crystal packing interactions, particularly in the P1 and latch regions (Fig. S5). Therefore, it is equally important to understand the underlying temporal information of these spatial changes. We used PVM to observe the first stage of the SSPT by monitoring changes in crystal birefringence under cross-polarized light (Fig. 4 ▸).

Importantly, a ligand concentration of 10 m*M* was used to match that of the TRX experiments. The onset of the SSPT is observable as waves of decreasing intensity of birefringence that originate primarily from one edge of the crystal at a defect [Fig. 4 ▸(*a*), green arrow]. The slow build-up of [apo1/IB·Ade], the duration of which is dependent on the ligand concentration, leads to thermal expansion and accumulated crystal strain. The phase transition is then triggered by structural and lattice changes associated with conformational switching, resulting in the sudden and stochastic release of energy, which is evident optically by birefringence and physically in the form of crystal motion and stress fractures [Fig. 4 ▸(*a*)]. The physical manifestations of energy release are analogous to those observed in ‘jumping crystals’ of small molecules (Commins *et al.*, 2016 ▸; Skoko *et al.*, 2010 ▸). As time goes on, and the crystal lattice stabilizes, many of the fractures anneal and crystal birefringence gradually increases [Figs. 4 ▸(*a*) and 4(*b*)].

**Figure 5:**
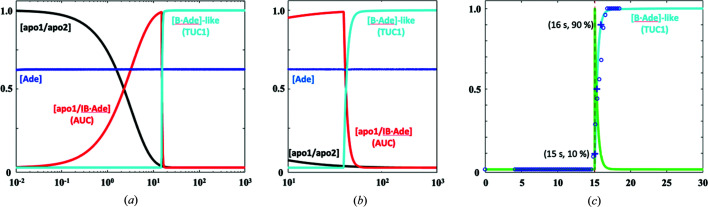
(*a*) Semi-quantitative kinetic profile of conformation species in the crystals based on the birefringence intensity measurements using a two-step model. (*b*) Expanded time range in (*a*) showing the coexistence of AUC (red) and TUC1 (cyan). (*c*) Experimentally measured conversion fraction of [B·Ade]-like (blue circles) versus time superimposed with the time course of the first lattice transition (cyan). The first derivative (green) is also shown, which peaks at 15.2 s, the center of the T1 transition. The time points of [B·Ade]-like at 10 and 90% are marked.

For temporal measurements, we selected a region of interest (80 × 80 pixels, 2 × 2 µm) at the center of the crystal that is approximately equal to the diameter (1–2 µm) of the X-ray beam used in our XFEL experiments. The intensity of crystal birefringence was measured for this ROI and plotted as a function of time [Fig. 4 ▸(*b*)]. Plotting the first-derivative of the intensity curve reveals three distinct peaks (T1, T2 and T3) [Figs. 4 ▸(*c*) and 4(*d*)], which occur much earlier than in previous experiments using larger riboA crystals and lower concentration of ligand (Ramakrishnan, Stagno, Conrad *et al.*, 2021 ▸). The first stage of transition (T1), which is centered around 15.2 s, is expected to comprise the ligand-induced conformational changes and is, therefore, the primary area of interest for the purpose of this study (Fig. 4 ▸). The meaning of T2 and T3 requires further investigation. Using our previously reported kinetic model (Stagno *et al.*, 2017 ▸), we simulated the kinetics of the T1 AUC–TUC1 transition in the PVM experiment (Fig. 5 ▸, supporting information):[Chem scheme1] 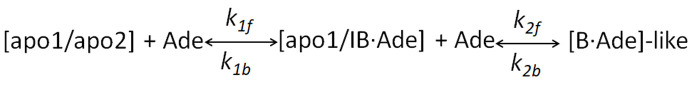


Given that the sharp T1 transition from the intensity curve is 90% complete within 1 s of onset, and the forward transition is unidirectional, the kinetics of T1 were simulated to best fit the experimental profile (Fig. 5 ▸), with rate constants *k*_1*f*_ and *k*_2*f*_ of 3.05 × 10^−5^ and 1.4 × 10^−4^, respectively. Notably, the rate-limiting step is the accumulation of [apo1/IB·Ade] followed by a sharp conversion to [B·Ade]-like. Such a kinetic characterization becomes possible only after an SSPT can be quantified using PVM in a transparent crystal.

The timing and speed of the AUC–TUC1 transition occur somewhat sooner and much more rapidly in our PVM experiments than what are observed by TRX at the same ligand concentration (Ramakrishnan, Stagno, Conrad *et al.*, 2021 ▸). In the PVM results, the T1 transition is complete by 16 s, whereas diffraction data acquired at 25 s showed only a small but emerging population of TUC1. There are many experimental factors that must be taken into consideration when comparing the results of these two methods. The first major difference lies in how mixing was performed in each case. In PVM, a small number of isolated crystals are rapidly saturated with ligand-containing buffer through diffusive mixing in a wide dish. In the mix-and-inject TRX experiments, however, the ligand buffer and an equal volume of an extremely dense and viscous slurry of microcrystals (10^9^–10^10^ ml^−1^) are fed through PEEK tubing into a 3D-printed microfluidic mixer. Secondly, the mix-and-inject method utilizes two independent pumps (one for sample, and one for ligand) upstream of the mixer, which often exhibit fluctuating pressures and flow-rates due to various factors such as clogging. Most importantly, however, since the transition was shown by AFM and PVM to be synchronous for a 2 × 2 µm ROI, the diffraction volume of each individual crystal illuminated by a 1–2 µm beam of X-rays should be mostly uniform, given that the largest face of the plate-like crystals is almost perpendicular to the incident beam.

In order to mitigate any effects of diffusive mixing on the interpretation of PVM results, we conducted a parallel experiment using a crystal presoaked with a photo-caged adenine ligand (Fig. S6). Here, the binding reaction and SSPT are triggered upon photo-release of adenine by exposing the crystal to a UV (365 nm) LED pulse. Although the timing of T1 in the photo-caged experiment (12.0 s) is quite similar to that in the mixing experiment (15.2 s), the subsequent T2 and T3 transitions occur much more quickly (T2: 12.5 versus 20.1 s; T3: 13.1 versus 23.4 s). Therefore, the relationship between the timing of the transitions in these two experiments is not linear. Additional studies may further characterize the differences observed using these two methods. Most likely, however, the diffusionless and virtually instant saturation of riboA molecules in the photo-caged experiment may produce a more uniform SSPT by aiding in the synchronization of conformational changes at the start of the transition, and subsequently throughout. This approach may prove advantageous in future studies, particularly X-ray diffraction experiments, for elucidating SSPT mechanisms with higher precision.

In riboA crystals, where large conformational changes are involved, the phase transition, generally speaking, can only occur via two possible pathways: (1) disordering of molecules into a liquid phase, followed by new lattice formation; or (2) cooperative motions of molecules which allow the structural integrity of the crystal to be maintained. Through direct real-time visualization of riboA crystals by both AFM and PVM, our results clearly demonstrate that structural changes and molecular rearrangements are accommodated by the crystalline environment such that an ordered solid phase is maintained throughout. This feature of the SSPT is critical for structure determination of intermediate conformations, making it possible to determine a molecular movie of a large conformational switch. To do so, however, requires temporal information for how and when the transitions occur, and the number of intermediate lattices that may be involved. Such analyses for defining the time-window of conformational changes are critical for informing future TRX studies using an XFEL. As we now know that the transition from AUC to TUC1 encompasses the full extent of ligand-induced conformational changes relevant to aptamer switching, the knowledge gained from these experiments allows us to focus our data acquisition on the time regime during which those changes occur.

## 4. Conclusions   

To our knowledge, this study represents the first report elucidating the mechanistic interplay between the conformational changes that drive an SSPT in a biomacromolecular crystal and the intermolecular lattice contacts associated with each stage of the transition. Although some of the packing interfaces are conserved in all three phases, there are distinct differences in the types (*e.g.* hydrogen bonds, VDW) and extent of interactions, as well as the orientations of molecules to achieve them. The conformational changes are also restricted by lattice energies and confined RNA conformational pathways (Bailor *et al.*, 2010 ▸, 2011 ▸), in which lattice order is maintained, even to the atomic level. This is in contrast to other examples of SSPTs that can involve different transition pathways. The most recent studies in nonbiological systems show that SSPTs triggered by heating or photons can occur via a metastable liquid phase (Peng *et al.*, 2015 ▸; Smets *et al.*, 2020 ▸; Pogatscher *et al.*, 2016 ▸; Mohammadi *et al.*, 2018 ▸; Taniguchi *et al.*, 2019 ▸). In crystals of linear amino acids, synchronous cooperative motion of molecules was shown to be only one of many possible transition mechanisms (Smets *et al.*, 2020 ▸). The first-order (continuous) or second-order (discontinuous) classificaitons of SSPTs by Ehrenfest have since been challenged (Mnyukh, 2009 ▸). Mnyukh argued that all SSPTs are first order in nature and follow a mechanism of continuous nucleation and growth (molecule-by-molecule). Similar to the study by Smets *et al.* (2020 ▸), however, our results demonstrate that the nucleation-and-growth process of the SSPT in riboA crystals is not mutually exclusive to the cooperative motions of subdomains of molecules. Importantly, the mechanism of these changes is not theoretical, but is directly observable and measurable using the combination of PVM, AFM and TRX. This more complex phenomenon of nucleation and growth coupled with molecular synchronization may be a particular distinction in crystals of biomolecules, which involve weaker and much more dynamic interactions in crystals with much higher solvent content. Despite its complexity, the SSPT observed in riboA crystals is reversible. The development of ‘switchable’ materials using external stimuli is of great scientific interest, not only for materials science and technology but also for biology and pharamaceuticals. Therefore, expanding such research to include SSPTs of biomacromolecular crystals and their mechanisms could have broad and profound implications.

## Supplementary Material

Supporting information file. DOI: 10.1107/S2052252521004826/lq5040sup1.pdf
