# Supplementary material for: The mechanism driving a solid–solid phase transition in a biomacromolecular crystal
Source: IUCrJ. 2021 Jun 17;8(Pt 4):655–64. doi: 10.1107/S2052252521004826 (PMC8256710; doi:10.1107/S2052252521004826)
Supplement: Supplementary file 1 [file m-08-00655-sup1.pdf]

IUCrJ

Volume 8 (2021)

Supporting information for article:

The mechanism driving a solid-solid phase transition in a biomacromolecular crystal

Saminathan Ramakrishnan, Jason R. Stagno, William F. Heinz, Xiaobing Zuo, Ping Yu and Yun-Xing Wang

S1. Kinetics of the first stage of the phase transition (AUC to TUC1)

The detailed molecular mechanism of the ligand-triggered conformational switch, and the timeline of the concurrent first lattice change from AUC to TUC1, allow for a semi-quantitative temporal kinetics description of T1 (Fig. 5). If one assumes the crystal under consideration as a homogeneous system (with respect to “bulk” kinetics), (<sgmlfi>\partial I{\left({x,y,t} \right)_i}/\partial {t_i})</sgmlfi>) over all p(xy)i of the sampled area (80 x 80 pixels^2) vs. time reveals the time course of the transition. Based on our kinetics study in solution reported previously (Stagno et al., 2017), we found that the following model is sufficient to account for the kinetics of the ligand-triggered conformational switch:

$$[\text{apo1/apo2}] + \text{Ade} \underset{k_{1b}}{\overset{k_{1f}}{\longleftrightarrow}} [\text{apo1/IB·Ade}] + \text{Ade} \underset{k_{2b}}{\overset{k_{2f}}{\longleftrightarrow}} [\text{B·Ade}]\text{-like} \qquad \text{Eq. 1}$$

where $k_{1f} \sim 3.05\text{x}10^{-5}$ and $k_{2f} \sim 1.4\text{x}10^{-4}$. k_{1b} and k_{2b} are set to zero under the condition of unidirectional phase transition in crystal. The multivariance kinetic equations were solved numerically using a MatLab script to obtain the rate constants above and resulted in a kinetic profile consistent with the explicit observations from our polarized video microscopy (PVM), atomic force microscopy, and with our previous solution kinetics model (Stagno et al., 2017, Ramakrishnan et al., 2020). Given the unit cell parameters and number of the molecules per unit cell, the concentration of [apo1/apo2] in AUC is ~16 mM. The ligand concentration was 10 mM for the PVM kinetics experiments. The results of the simulation describe the T1 phase transition as follows. T1 starts at ~15.2 s after mixing with ligand, where the molecules begin to change conformation. The first derivative of T1 is centered between 15 and 16 s, where ~50% of [apo1/IB·Ade] converts to [B·Ade]-like. 90% of the T1 transition completes in < 1 s. As two molecules of [IB·Ade] or [B·Ade]-like cannot be accommodated sterically in the AUC lattice without expansion of the unit cell and disruption of crystal contacts, the resultant lattice upon transition is TUC1.

Table S1 The buried surface areas (Å2) between molecular interfaces in the ab or ac plane.

	AUC	TUC1	BUC
ab plane	1028	1053	1079
ac plane	742.5	619.5	792
Total	1770.5	1672.5	1871

Table S2 Unit cell dimensions of forward and reverse phase transitions (AUC topo 1 , BUC topo 1 and AUCrev topo) in *ac* crystal at 35 ° C

	a(Å)	c(Å)	β(°)
AUC topo 1	50.1	93.6	93.2
BUC topo 1	49.8	157.3	90.6
AUCrev topo	51.1	94.2	91.4

Table S3 Unit cell parameters for AUC, TUC1, and BUC, derived from X-ray diffraction data.

ac plane	a(Å)	c(Å)	β(°)
AUC	48.3	93.0	94.3
TUC1	50.3	78.5	90
BUC	50.3	155.6	90
ab plane	a(Å)	b(Å)	ɣ (°)
AUC	48.3	47.0	90
TUC1	50.3	25.2	90
BUC	50.3	25.3	90

Table S4 Unit cell parameters for AUC, TUC1, and BUC, derived from AFM data (ac or ab plane).

ac plane	a(Å)	c(Å)	β(°)
AUC	49.7	94.5	93.1
TUC1	49.8	78.2	90.2
BUC	49.2	158.2	89.5
ab plane	a(Å)	b(Å)	ɣ (°)
AUC	49.1	46.0	90.4
TUC1	50.1	24.3	90.6
BUC	51.2	25.1	90.8

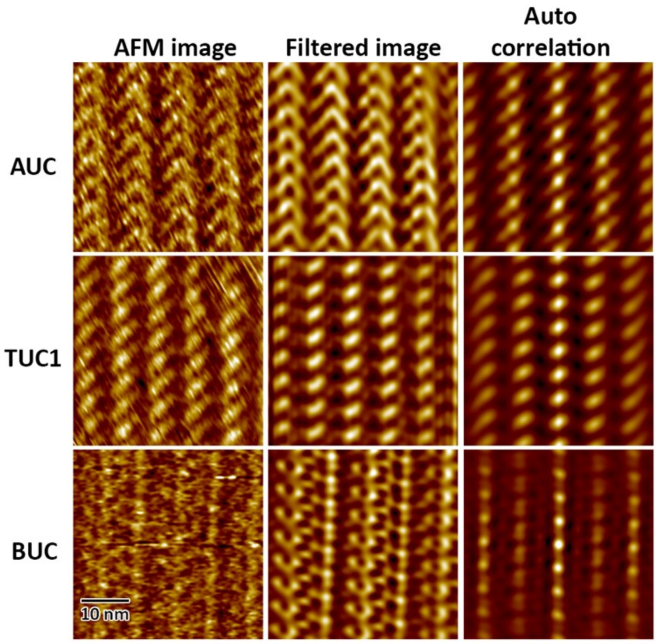

Figure S1 The AFM image, filtered and autocorrelation images of the AUC, TUC1, and BUC *ac*-planes. The corresponding unit cell dimensions are listed in **Table S3**. The AFM images are 40 X 40 nm^2 in size and 0.5 nm in height (dark to light color scale).

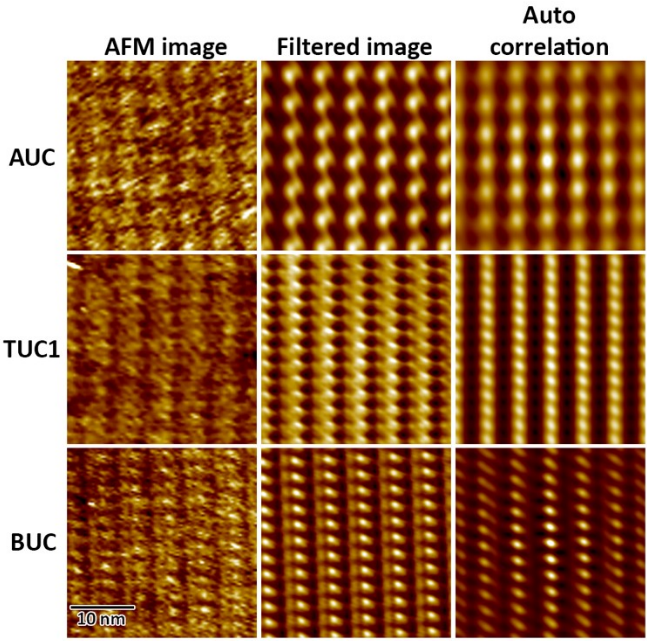

Figure S2 The AFM image, filtered and autocorrelation images of the AUC, TUC1, and BUC *ab*-planes. The corresponding unit cell dimensions are listed in Table S3. The AFM images are 30 x 30 nm^2 in size and 0.5 nm in height (dark to light color scale).

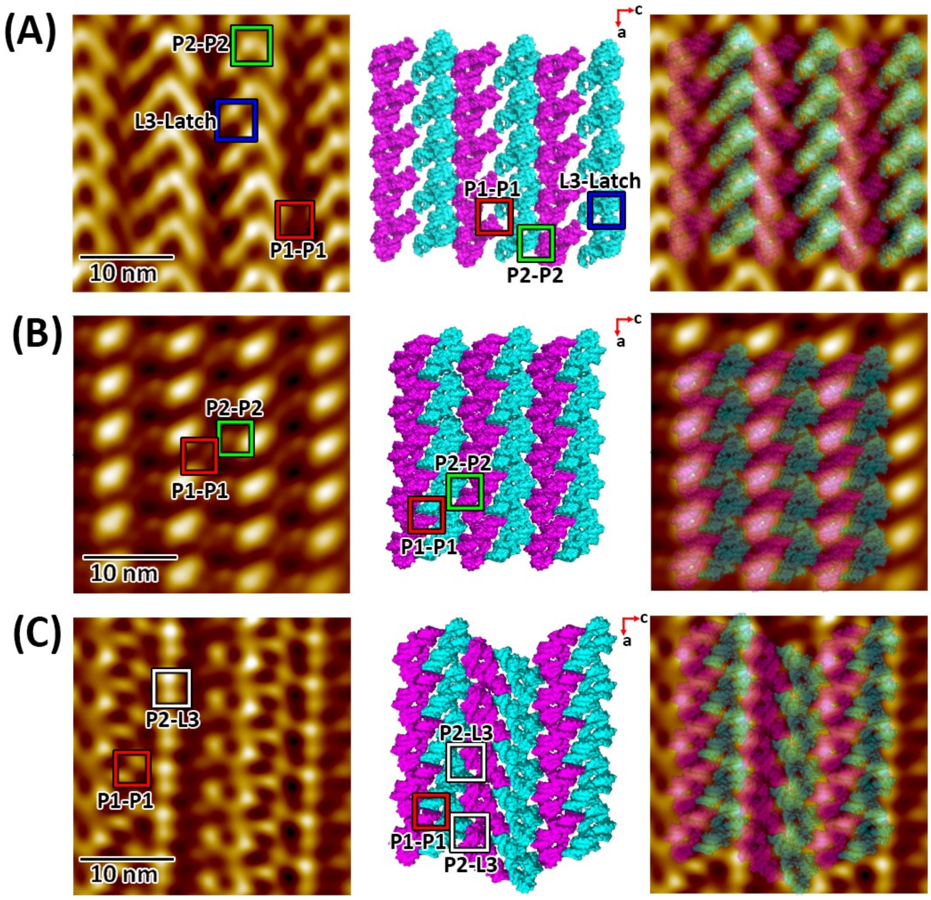

Figure S3 The filtered images (left) and single layer of molecules derived from the crystal structures (middle) of (*A*) AUC, (*B*) TUC1, and (*C*) BUC *ac*-planes. The right column of images shows the superimposition of the riboA molecules, as they exist in their respective crystal structures, onto the AFM images. The molecular interfaces in each phase are highlighted with colored boxes. The AFM images are 30 x 30 nm^2 in size and 0.5 nm in height (dark to light color scale).

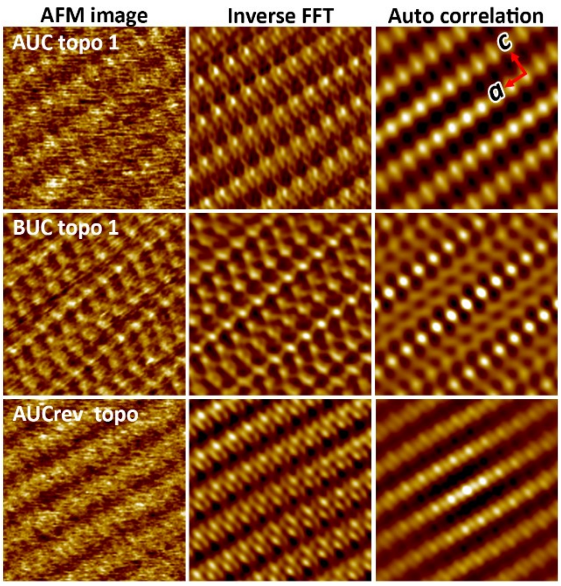

Figure S4 AFM images, filtered images and corresponding autocorrelation functions of forward and reverse SSPT in *ac*-face riboA crystals. The forward SSPT from AUC to BUC in the presence of 50 μM adenine ligand at 15 °C was reversed to AUCrev crystal structure after extensive washing with ligand free buffer at 35 °C. The unit cell dimensions of all the stages are listed in Table S4. AFM images are 50 x 50 nm^2 in size and 0.5 nm in height (dark to light color scale)

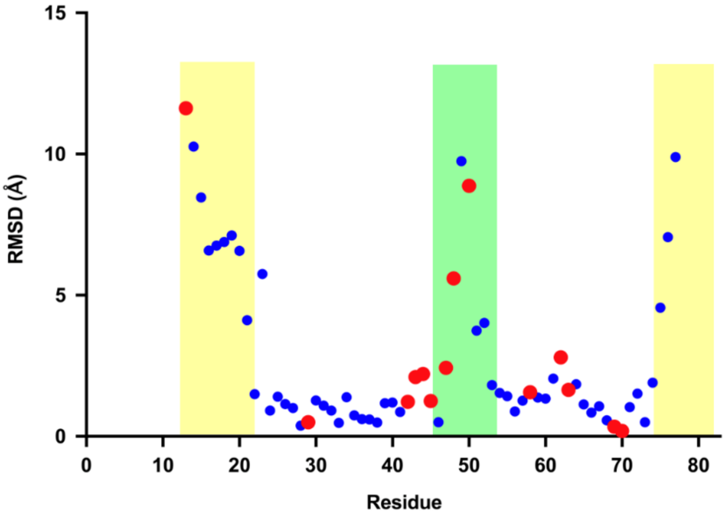

Figure S5 Plot of RMSD per residue after structural superposition of AUC (apo2) and TUC1. Structurally conserved residues 25-31, 39-45, 54-59, and 67-72 were used in the all-atom alignment. Residues 78-83 were omitted since those residues are disordered in the AUC model. Residues involved in *ac*-crystal-packing interfaces are labeled (red circles). P1 and Latch regions are shaded in yellow and green, respectively.

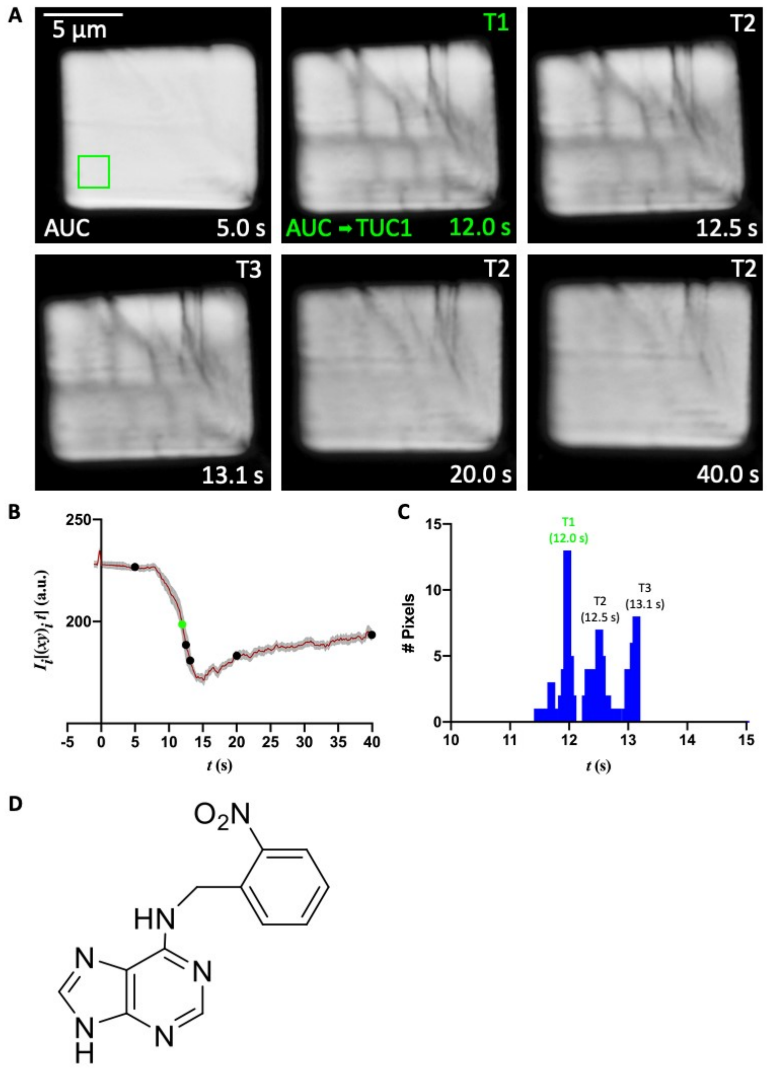

Figure S6 (*A*) Polarized video microscopy images of a riboA crystal (12 µm x 10 µm) soaked with 10 mM photocaged adenine (pcADE), and illuminated with a 2 s LED pulse (4.5 A) at 365 nm. The video was recorded with an exposure time of 300 ms. (*B*) The averaged intensity of crystal birefringence for each pixel for the selected area (80 x 80 pixels^2, green square) plotted vs. time. (*C*) Histogram of the time values of the first-derivative peaks of spatially averaged (10 x 10 pixels^2) pixels, revealing T1, T2, and T3. Time zero was taken to be the time of the LED pulse, which is represented by the intial spike in the intensity curve in *B*. (*D*) Chemical structure of the pcADE compound used in the PVM experiment.
